# Acral Eruptive Syringoma: An Unusual Presentation with Misdiagnosis

**DOI:** 10.1155/2020/5416285

**Published:** 2020-01-11

**Authors:** Fatemeh Mohaghegh, Akramsadat Amiri, Farahnaz Fatemi Naeini, Parvin Rajabi, Maryam Soltan

**Affiliations:** ^1^Department of Dermatology, School of Medicine, Isfahan University of Medical Sciences, Isfahan, Iran; ^2^Department of Pathology, School of Medicine, Isfahan University of Medical Sciences, Isfahan, Iran

## Abstract

Eruptive syringoma is a rare variant of syringoma presenting with skin-colored or slightly pigmented papules mostly before or during puberty. In this report, we presented a rare case of eruptive syringoma in a 30-year-old woman. She exhibited multiple skin lesions in dorsal areas of her both hands, developed from the age of 15.

## 1. Introduction

Syringomas are well known to be benign adnexal tumors detected most commonly in periorbital skin areas of middle-aged women [1]. Eruptive syringoma is a rare variant of syringoma which appears in large numbers as multiple skin-colored or slightly pigmented papules on the anterior chest, neck, upper abdomen, axillae, and periumbilical region before or during puberty [2, 3]. A few number of syringoma cases with predominantly acral distribution have been reported previously [4, 5]. In this paper, we present a rare case of eruptive syringoma localized in dorsal areas of both hands in a 30-year-old woman.

## 2. Case

We presented a 30-year-old woman with eruptive syringoma of about 15 years duration, who was referred to the dermatology clinic of Alzahra hospital, Isfahan, Iran. Lesions started from the dorsal side of the patient's right hand without presenting other symptoms such as pruritus, burning sensation, inflammation, or erythema. The number of lesions increased following the epilation of the area for several times and no improvement was observed with the use of local corticosteroids. As a result, she stopped the epilation of her dorsal hand area. The patient refused to do a skin biopsy upon the suggestion of a dermatologist.

**Figure 1:**
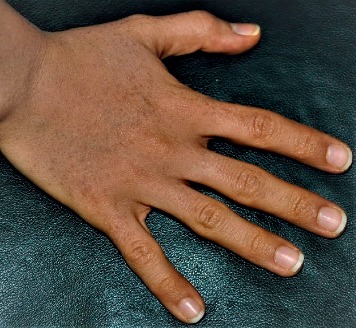
Multiple, erythematous, flat-topped papules located on dorsal areas of both hands.

At the age of 26 the patient was visited by an expert dermatologist with complaints about the expansion of the lesions to the dorsal areas of her left hand and underwent trichloroacetic acid (TCA) therapy twice with one year gap with suspicion of plane warts. Physical examinations in our clinic showed multiple erythematous, flat-topped papules located on dorsal areas of her hands varying from 1 to 3 mm in size (Figure 1).

**Figure 2:**
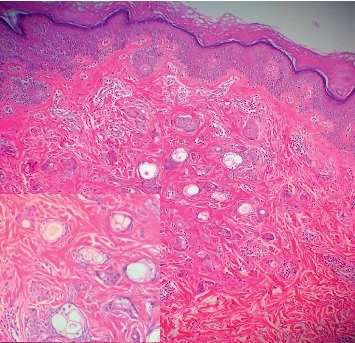
Skin biopsy and pathological studies revealing multiple nests of cells with pale cytoplasm positioned within sclerotic stroma.

Lesions were symmetrical, and Darier's sign was negative. The family history was unremarkable, and there was no previous history of skin disorders. Laboratory test results were normal, and the patient underwent biopsy of skin lesions. Finally, the diagnosis of eruptive syringoma was made based on both clinical and histological findings. In histological sections, multiple nests of cells were seen with pale cytoplasm positioned within sclerotic stroma. Many nests show central ductal differentiation with a compact eosinophilic cuticle. The epidermis is normal; however, numerous tubular structures are embedded in a dense stroma in dermis, and ducts are lined by two rows of epithelial cells. Some of them have comma-like tails (tadpoles) (Figure 2).

After establishing the diagnosis, we recommended her some destructive modalities like cautery or radiofrequency for treating these disfiguring lesions, but the patient refused to undergo any treatment.

## 3. Discussion

Eruptive syringoma is known to be a rare variant of syringoma with nonspecific clinical findings. The diagnosis of eruptive syringoma usually is based on performing skin biopsies and histological examinations. Clinical diagnosis of the disease is almost difficult due to its similarity to other skin diseases such as acne vulgaris, sebaceous hyperplasia, lichen planus, urticaria pigmentosa, eruptive vellus hair cysts, sarcoidosis, warts, and lentigo [6–8]. The same as our case, former studies have reported a misdiagnosis of eruptive syringoma with warts. The results of these studies have shown that the treatment of suspicious lesions resulted in no improvement and final diagnosis of disease was made by histopathological examinations [1, 9].

No prolonged morbidity or mortality due to eruptive syringoma has been reported [7]. However, there are a variety of therapeutic approaches mostly for cosmetic purposes including, surgical excision, chemical peeling, topical atropine, topical and oral retinoids to manage the condition [10]. Yet, most of these treatment options are unsatisfactory and there is the risk of lesions' recurrence, scarring, and pigmentation after treatment [11].

Although eruptive syringoma commonly occurs before or during puberty, it could be diagnosed in any period of life. Our case developed skin lesions after puberty, but the definite diagnosis of the disease occurred at the age of 30. Similarly, Sakiyama et al. reported eruptive syringoma in two females aged 43 and 35 years old [12]. The possible explanation for the late diagnosis of the disease in the present case is her refusal to perform skin biopsy. Finally, our study shows that clinicians should not solely rely on clinical findings for the diagnosis of eruptive syringoma and histopathological findings should also be taken into account.
